# The complete mitochondrial genome of parasitic nematode *Camallanus cotti*: extreme discontinuity in the rate of mitogenomic architecture evolution within the Chromadorea class

**DOI:** 10.1186/s12864-017-4237-x

**Published:** 2017-11-02

**Authors:** Hong Zou, Ivan Jakovlić, Rong Chen, Dong Zhang, Jin Zhang, Wen-Xiang Li, Gui-Tang Wang

**Affiliations:** 1 Key Laboratory of Aquaculture Disease Control, Ministry of Agriculture, and State Key Laboratory of Freshwater Ecology and Biotechnology, Institute of Hydrobiology, Chinese Academy of Sciences, Wuhan, 430072 People’s Republic of China; 2 Bio-Transduction Lab, Wuhan Institute of Biotechnology, Wuhan, 430075 People’s Republic of China; 3 University of Chinese Academy of Sciences, Beijing, 100049 People’s Republic of China

**Keywords:** Gene order rearrangement, Gene duplication, tRNA duplication, Incomplete tRNA set, tRNA remolding, TDRL, Pseudogenes, Mitochondrial phylogenomics, Spirurida, Camallanina

## Abstract

**Background:**

Complete mitochondrial genomes are much better suited for the taxonomic identification and phylogenetic studies of nematodes than morphology or traditionally-used molecular markers, but they remain unavailable for the entire Camallanidae family (Chromadorea). As the only published mitogenome in the Camallanina suborder (Dracunculoidea superfamily) exhibited a unique gene order, the other objective of this research was to study the evolution of mitochondrial architecture in the Spirurida order. Thus, we sequenced the complete mitogenome of the *Camallanus cotti* fish parasite and conducted structural and phylogenomic comparative analyses with all available Spirurida mitogenomes.

**Results:**

The mitogenome is exceptionally large (17,901 bp) among the Chromadorea and, with 46 (pseudo-) genes, exhibits a unique architecture among nematodes. Six protein-coding genes (PCGs) and six tRNAs are duplicated. An additional (seventh) tRNA (Trp) was probably duplicated by the remolding of tRNA-Ser2 (missing). Two pairs of these duplicated PCGs might be functional; three were incomplete and one contained stop codons. Apart from Ala and Asp, all other duplicated tRNAs are conserved and probably functional. Only 19 unique tRNAs were found. Phylogenomic analysis included Gnathostomatidae (Spirurina) in the Camallanina suborder.

**Conclusions:**

Within the Nematoda, comparable PCG duplications were observed only in the enoplean Mermithidae family, but those result from mitochondrial recombination, whereas characteristics of the studied mitogenome suggest that likely rearrangement mechanisms are either a series of duplications, transpositions and random loss events, or duplication, fragmentation and subsequent reassembly of the mitogenome. We put forward a hypothesis that the evolution of mitogenomic architecture is extremely discontinuous, and that once a long period of stasis in gene order and content has been punctuated by a rearrangement event, such a destabilised mitogenome is much more likely to undergo subsequent rearrangement events, resulting in an exponentially accelerated evolutionary rate of mitogenomic rearrangements. Implications of this model are particularly important for the application of gene order similarity as an additive source of phylogenetic information. Chromadorean nematodes, and particularly Camallanina clade (with *C. cotti* as an example of extremely accelerated rate of rearrangements), might be a good model to further study this discontinuity in the dynamics of mitogenomic evolution.

**Electronic supplementary material:**

The online version of this article (doi: 10.1186/s12864-017-4237-x) contains supplementary material, which is available to authorized users.

## Background

Metazoan invertebrate phylum Nematoda is likely to comprise as much as 90% of all living multicellular organisms [1–3]. Because of the parasitic lifestyles of many nematodes, which cause numerous human diseases and large financial losses to agriculture and livestock rearing, as well as their use as biodiversity indicators and model organisms (e.g. *Caenorhabditis*), nematodes have attracted ample scientific attention [4, 5]. Prior to the application of molecular data, nematode taxonomy and phylogeny relied on a very limited number of morphological characters and ecological features. This, along with the absence of informative fossil records [5, 6] and pervasive convergent evolution, has resulted in very poorly resolved and often inconsistent classification systems [2, 5, 7]. Thus, molecular data are much better suited for identification and phylogenetic studies of nematodes. The most often used genetic marker for this type of studies in nematodes is 18S rRNA (or nSSU) [1, 2, 5]. However, since the number of nematode taxa will soon exceed the number of nucleotides in this gene, it can be safely argued that even the theoretical resolving power of this approach is insufficient for the task [3]. Thus a marker with much higher resolving power shall have to be adopted by future studies. Complete mitogenomes appear as a strong candidate, as they can provide a phylogenetic resolution superior to the traditionally used molecular markers and precise divergence date estimates, and thus are becoming the marker of choice for the resolution of taxonomic controversies [2, 8–13].

The mitochondrion is a eukaryotic organelle descended about 1,000,000,000 years ago from an α-proteobacterium [14, 15], whose major function in the organism is energy production. Mitochondria contain their own genomes with a modified genetic code, which are usually very compact (no introns, little intergenic DNA, and most genes show signs of selection for small size) and highly conserved in terms of gene content and organisation [10, 16–18]. Mitogenomes of nematodes, however, are characterized by relatively frequent gene rearrangements [2, 7, 13], unique initiation codons [19, 20], fast nucleotide substitution rate, unconventional tRNAs [10], and sometimes even unique organisation [21, 22], which makes them a promising model for studying the mechanisms of mitochondrial gene rearrangements and genome architecture evolution [9]. So far, however, the main obstacle to their broad application has been a limited availability of sequenced mitochondrial genomes. Even though the number of complete mitogenomes deposited in public databases has grown exponentially during the last few years, many taxonomic categories remain poorly or not at all represented.

Among the non-represented taxa is the entire Camallanoidea superfamily (Camallanina suborder, Spirurida order, Chromadorea class). Spirurida order is composed of Spirurina and Camallanina suborders, the latter of which contains only Camallanoidea and Dracunculoidea superfamilies. Camallanidae (the only Camallanoidea family) are almost globally-distributed gastrointestinal parasites of poikilothermic vertebrates [1]. Only two *Camallanus* species parasitising Chinese freshwater fish are currently recognised: *C. cotti* and *C. hypophthalmichthys* [23]. The former, *C. cotti* (Fujita 1927; synonyms: *C. zacconis* Li 1941 and *C. fotedari* Raina & Dhar 1972) parasitises a large number of fish species, mostly belonging to Cypriniformes, Siluriformes and Perciformes orders [24]. Although native to Asia, as a result of the trade of ornamental fishes and the introduction of various poeciliids for mosquito control, it has become almost globally distributed during the last few decades [24, 25]. Its cosmopolitan dispersal, relatively high pathogenicity [24] and exceptional life history flexibility [25] have garnered ample scientific attention.

Single molecular markers, such as internal transcribed spacer of ribosomal DNA, have been employed to infer the phylogenetic relationships of Camallanidae [1, 23], but due to previously described limitations of this approach, phylogeny of this family and the entire Camallanina clade is poorly understood. Sequencing of the complete mitochondrial genome of *C. cotti*, and the associated phylogenomic analysis using all available mitogenomes belonging to Spirurida order, are meant to address this problem. More importantly, as both mitochondrial genomes that are currently available for the entire Camallanina suborder, *Philometroides sanguineus* [26] and *Dracunculus medinensis* (unpublished), exhibit unique gene orders [26, 27], we hypothesised that this mitogenomic architecture might be idiosyncratic to the entire Camallanina suborder. To establish whether the unique Dracunculoidea architecture is shared with the sister superfamily Camallanoidea, and thus contribute to the understanding of the evolution of mitochondrial genomic architecture of nematodes, we have sequenced and characterised the mitogenome of *C. cotti* and compared it structurally to the available Spirurida mitogenomes.

## Results and discussion

The mitochondrial genome of *C. cotti* possesses a large number of duplicated genes, which makes it structurally unique among the nematodes. The mitogenome is accessible from the GenBank under the accession number MF580344. To make sure that these duplicated sequences are not artefacts from our own PCR amplification or *numt*s [28], we have extracted DNA from an additional *C. cotti* specimen and amplified, sequenced and assembled its entire genome anew. The sequences were identical, apart from the minor variations expected among individual mitogenomes [29], thus excluding the possibility of a mistake in the sequencing and assembly process. Unfortunately, these two nematodes were obtained from the same fish specimen, so we cannot directly exclude the possibility of this unique architecture being a lineage-specific phenomenon. If identical, or very similar, architecture is not observed in other Camallanidae species in the future, it would be of interest to sequence another mitogenome belonging to this species to confirm that the architecture is species-specific.

### Taxonomic identity and phylogeny

Following proposed guidelines for validation of new mitogenomes [30], along with a phylogenetic analysis using almost the entire mitogenomic sequence, we have also conducted a barcoding identification assessment using all 99 Camallanidae *cox1* sequences available in the BOLD database [31]. The queried sequence was nested within the monophyletic *Camallanus cotti* clade (Additional file 1).

**Fig. 1:**
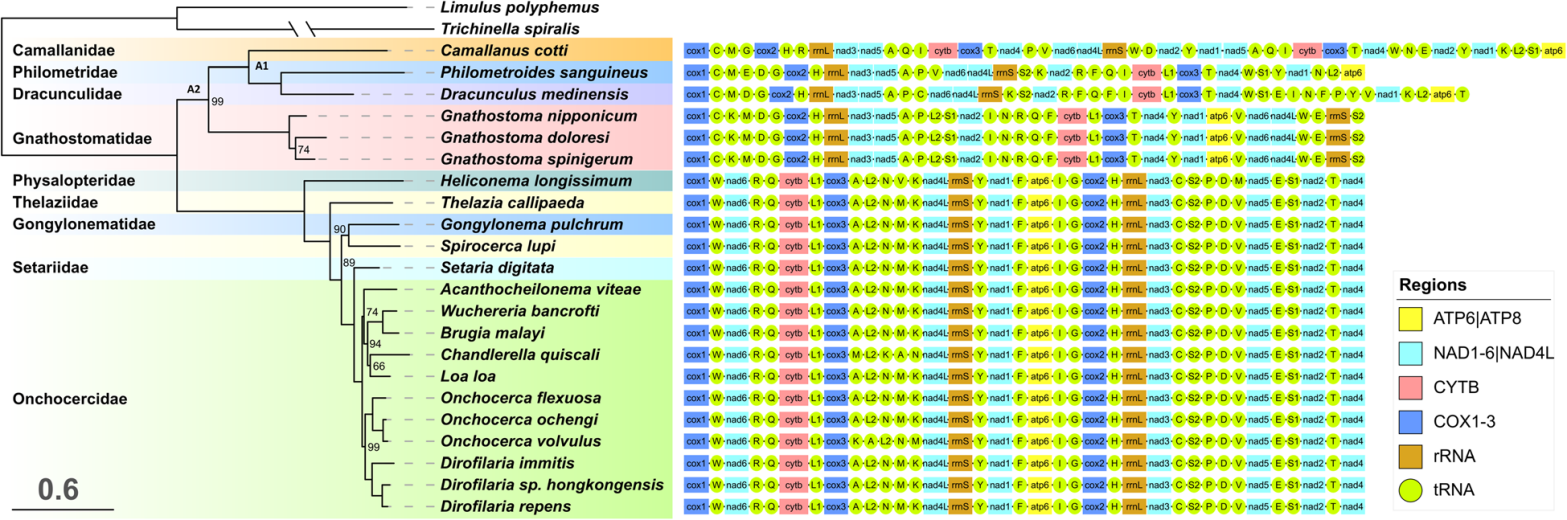
Phylogeny and mitogenomic architecture of the Spirurida order. Phylogenetic dendrogram constructed using gene sequences of 22 Spirurida mitochondrial genomes. Maximum likelihood and Bayesian inference approaches produced identical topologies, so only the former is shown. *L. polyphemus* and *T. spiralis* are outgroups. Scale bar corresponds to the estimated number of substitutions per site. Only the bootstrap values below 100 are shown. Mitogenomic architecture is shown to the right of the corresponding sequences. A1 and A2 are ancestral nodes. GenBank accession numbers are available in Table 1

The two approaches (maximum-likelihood and Bayesian inference) used in this study to estimate the phylogenetic position of *C. cotti* within the Spirurida clade produced identical dendrogram topologies, so only the former is shown in Fig. 1. Statistical support values were very high, particularly in the BI analysis, where all posterior probability values were 1.0. In (partial) agreement with previous studies using both nSSU rRNA and mitochondrial phylogenomics approaches [1, 5, 27, 32]. Our results indicate that Spirurida order is divided into both morphologically [1] and genetically clearly defined Spirurina suborder, here comprised of (Physalopteridae + (Thelaziidae + (Gongylonematidae + (Setariidae [paraphyletic] + Onchocercidae)))), and a monophyletic clade comprising (Gnathostomatidae + (Camallanidae + (Dracunculidae + Philometridae))), henceforth referred to as Camallanina suborder (Fig. 1, Table 1). Sister group relationship between the Camallanoidea superfamily (Camallanidae family) and Dracunculoidea superfamily (Philometridae and Dracunculidae in this study) is well-established [1, 5, 33, 34]. Paraphyly of Thelaziidae family, and particularly the problematic taxonomy of *Spirocerca* and *Thelazia* genera was also observed before [1, 5, 27, 35]. A very similar topology of Spirurina clade, including the sister group relationship between Spirocerca and Gongylonema genera, was produced before using a smaller number of mitogenomes [32, 36]. Therefore it can be concluded with confidence that *Spirocerca lupi* is closely related to *Gongylonema pulchrum* and that it belongs to Gongylonematidae family.

**Table 1:** Spirurida mitogenomes used for comparative and phylogenetic analyses in this study

Species name	Family	Accession No.	Reference
Camallanus cotti	Camallanidae	MF580344	this study
Dracunculus medinensis	Dracunculidae	NC_016019	unpublished
Philometroides sanguineus	Philometridae	NC_024931	[26]^b^
Acanthocheilonema viteae	Onchocercidae	NC_016197	[112]
Brugia malayi		NC_004298	[113]
Chandlerella quiscali		NC_014486	[112]
Dirofilaria immitis		NC_005305	[62]
Wuchereria bancrofti		NC_016186	[112]
Loa loa		NC_016199	[112]
Dirofilaria sp. ‘hongkongensis’		NC_031365	[36]
Dirofilaria repens		NC_029975	unpublished
Onchocerca ochengi		NC_031891	unpublished
Onchocerca volvulus		NC_001861	[114]
Onchocerca flexuosa		NC_016172	[112]
Setaria digitata	Setariidae	NC_014282	[63]
Gnathostoma spinigerum	Gnathostomatidae	NC_027726	[37]
Gnathostoma doloresi		NC_032073	[38]
Gnathostoma nipponicum		NC_034239	[38]
Heliconema longissimum	Physalopteridae	NC_016127	[2]
Gongylonema pulchrum	Gongylonematidae	NC_026687	[103]
Spirocerca lupi	Thelazioidea	NC_021135	[35]
Thelazia callipaeda		NC_018363	[32]
Trichinella spiralis^a^	Trichinellidae	NC_002681	[50]
*Limulus polyphemus* ^a^	Limulidae	NC_003057	[104]

^a^indicates outgroup

^b^The reference for this sequence is about *Philometra carassii* (Nematoda: Philometridae). We presume that the name has been changed subsequently

Taxonomic position of the *Gnathostoma* genus, officially classified in the infraorder Gnathostomatomorpha of the suborder Spirurina [37], again proved to be contentious: in several studies using 18S rRNA, Gnathostomatomorpha formed a separate clade basal to all Spirurida [1, 4, 34], whereas in both studies reporting *Gnathostoma* mitogenomes [37, 38], phylogenomic analyses placed it within the Ascaridomorpha order. In our study, however, it formed a sister clade with (Camallanidae + (Dracunculidae + Philometridae)) within the Camallanina suborder. It is difficult to conclude which of these topologies are artefactual, as Ascaridomorpha were not included in our study. However, as Camallanina mitogenomes were not available for those two reports [37, 38], and as only amino acid sequences of protein-coding genes (PCGs) were used for their phylogenomic analyses, we suspect that the close relationship with Ascaridomorpha is an artefact likely to have been caused by long-branch attraction (LBA) [39, 40]. Amino acid sequences often exhibit too low variability in closely related species to allow a satisfactory level of phylogenetic resolution [11]. Furthermore, our results are much closer to the established phylogenetic position of Gnathostomatomorpha. Resolution of the intriguing taxonomic position of this infraorder thus warrants sequencing of further Camallanina mitogenomes, and inclusion of a much broader range of Nematoda families in future phylogenomic studies. An LBA artefact was also observed between the two outgroups used in this analysis: *Limulus polyphemus* (a basal arthropod) and *Trichinella spiralis* (a basal nematode), but it is not likely to have affected the topology of the Spirurida order.

### Genome size

At 17901 bp in size, the mitogenome of *C. cotti* is by far the largest among the available Spirurida mitogenomes (Additional file 2). It is also exceptionally large among the chromadorean nematodes, whose mitogenomes are usually in the range between 13 and 15 Kb [9, 10]. Enoplean nematodes exhibit a much stronger heterogeneity in mitogenome size, and sizes around and over 20 Kb are not uncommon [9, 10, 13]. Although duplicated protein-coding genes are common in mitogenomes of some metazoan groups [41, 42], major variations in mitogenome sizes can usually be attributed to differences in the length of noncoding regions, rather than variations in gene content [10, 16]. In agreement with this, the few known chromadorean mitogenomes larger than *C. cotti,* mostly found in plant-parasitic nematodes, possess abnormally lengthy non-coding regions that harbor tandemly repeated sequences [13, 43–45]. Large duplicated coding regions haven’t been observed in chromadorean mitogenomes so far. The only comparable duplications, containing PCGs, have been observed in the enoplean family Mermithidae [45, 46]. Thus, as a vertebrate-parasitic nematode exhibiting a large number of duplicated PCGs and tRNAs (Fig. 1), *C. cotti* is an exception not only among chromadoreans, but also among almost all nematodes.

### Genomic architecture

Most Nematoda mitogenomes contain 12 PCGs, as *atp8* gene is missing in all Spirurida and other more derived nematode groups [32], 22 tRNAs and two rRNAs, but the total number of genes is relatively variable (37.6 ± 2.9) [10]. Nevertheless, with 46 (complete or pseudo) genes (Table 2), *C. cotti* mitogenome is (again) exceptional. All of these genes are encoded on the same strand, which is typical for Chromadorean mitogenomes [9, 10, 13]. Disregarding the duplicated genes, the mitogenome possesses all of the standard 12 PCGs and both rRNAs. However, despite our best efforts, which included careful manual searches within all intergenic regions (IGR) in the mitogenome, we could only identify 19 of the standard 22 tRNAs, with Leu1^(CUN)^, Ser2^(AGN)^ and Phe missing.

**Table 2:** Organisation of the mitochondrial genome of *Camallanus cotti*

	Position			Codon				
Gene	Start	End	Size	Start	Stop	Anticodon	Strand	IG/O
12S	1	714	714				+	
tRNA-Trp-2	715	767	53			TCA	+	
tRNA-Asp	767	824	58			GTC	+	-1
pseudo-nad2	830	1640	811	ATT	T--		+	
tRNA-Tyr	1645	1698	54			GTA	+	4
pseudo-nad1	1701	2110	410				+	2
pseudo-nad5	2390	2760	371				+	279
pseudo-tRNA-Ala	2834	2893	60			TGC	+	73
tRNA-Gln	2883	2935	53			TTG	+	−11
tRNA-Ile	2934	2986	53			GAT	+	−2
cytb	2998	4081	1084	GTT	T--		+	11
cox3	4126	4887	762	ATT	TAA		+	44
tRNA-Thr	4883	4937	55			TGT	+	−5
nad4	4938	6170	1233	GTT	TAG		+	
tRNA-Trp	6170	6225	56			TCA	+	−1
tRNA-Asn	6226	6280	55			GTT	+	
tRNA-Glu	6282	6336	55			TTC	+	1
pseudo-tRNA-Asp	6337	6369	33			–		
nad2	6370	7180	811	ATT	T--		+	
tRNA-Tyr − 2	7189	7242	54			GTA	+	8
nad1	7243	8118	876	TTG	TAA		+	
tRNA-Lys	8117	8176	60			TTT	+	-2
tRNA-Leu2^(UUR)^	8177	8233	57			TAA	+	
tRNA-Ser1^(UCN)^	8234	8287	54			TCT	+	
atp6	8336	9002	667	TTG	T--		+	48
cox1	9091	10,644	1554	TTG	TAG		+	88
tRNA-Cys	10,646	10,700	55			GCA	+	1
tRNA-Met	10,704	10,757	54			CAT	+	3
tRNA-Gly	10,758	10,813	56			TCC	+	
cox2	10,814	11,489	676	TTG	T--		+	
tRNA-His	11,493	11,543	51			GTG	+	3
AT-rich	11,544	11,780	237				+	
tRNA-Arg	11,781	11,833	53			ACG	+	
16S	11,881	12,818	938				+	47
nad3	12,808	13,141	334	TTG	T--		+	−11
nad5	13,142	14,725	1584	TTG	TAG		+	
tRNA-Ala	14,750	14,803	54			TGC	+	24
tRNA-Gln − 2	14,808	14,860	53			TTG	+	4
tRNA-Ile-2	14,859	14,911	53			GAT	+	-2
cytb-2	14,909	16,007	1099	GTT	T--		+	−3
cox3–2	16,052	16,813	762	ATT	TAA		+	44
tRNA-Thr-2	16,809	16,863	55			TGT	+	−5
pseudo-nad4	16,879	17,121	243				+	15
tRNA-Pro	17,129	17,181	53			TGG	+	7
tRNA-Val	17,181	17,239	59			TAC	+	−1
nad6	17,238	17,673	436	TTG	T--		+	−2
nad4L	17,668	17,901	234	TTG	TAG		+	−6

IG/O: positive values indicate a non-coding intergenic segment, negative values indicate an overlap. Based on the hypothetical evolutionary history of genomic rearrangements (see Additional file 3 for details), we added a suffix ‘-2’ to the names of genes presumed to be copies of the original genes. Where sequence analysis indicated a loss of functionality, we added a prefix ‘pseudo-‘

In order to attempt to understand the evolutionary history of the unique mitogenomic architecture of *C. cotti*, we have compared it to other available Spirurida mitogenomes (Fig. 1). Gene order is almost perfectly conserved within the Spirurina suborder; minor exceptions are *Heliconema longissimum*, where tRNA-V and tRNA-M have switched places, and two species where minor rearrangements within the standard A-L2-N-M-K tRNA box can be observed: *Chandlerella quiscali* (M-L2-K-A-N) and *Onchocerca volvulus* (K-A-L2-N-M). As we suspected a possibility of an annotation artefact in *H. longissimum* (V and M), we have checked the two tRNAs: our results indicate that the genomic segment annotated as tRNA-Val [2] is not a functional tRNA. A deeper analysis of the entire mitogenome would be needed to determine the exact extent of its genomic architecture rearrangements.

In comparison to the Spirurina clade, genome architecture in the Camallanina clade is very different and very variable. A large number of gene rearrangements, including PCGs as well, can be observed, both between the two suborders and within the Camallanina clade. In terms of the order of PCGs (disregarding the tRNAs), three main patterns can be observed within the Camallanina clade: Gnathostomatidae pattern (perfectly conserved architecture among the three included species); Dracunculidae + Philometridae pattern exhibiting a transposition of the fragment containing *nad6*, *nad4l* and *12S rRNA* genes and a large number of tRNA rearrangements (a large number of tRNA rearrangements can be also observed between the two included species, *Philometroides sanguineus* and *Dracunculus medinensis*); and finally the unique Camallanidae pattern exhibiting a large number of rearrangements in the order of PCGs, as well as PCG duplications. Whereas the mitogenomic gene order in vertebrates is almost invariant, in invertebrates it is relatively variable; however, this is mostly due to variation in the number and location of tRNAs, whereas the order of PCGs and rRNAs is considered to be stable [41]. Therefore, Camallanina clade is an intriguing outlier.

### Characteristics of duplicated fragments of the mitogenome

To get a general idea about the origin and evolution of these duplicated fragments, we have compared the two *nad5-A-Q-I-cytb-cox3-T-nad4* fragments (*nad5-nad4* fragment henceforth). Because the two flanking genes (*nad5-nad4*) are not complete in both fragments (pseudogenes), only partial sequences of these two genes were included in the analysis. The upstream (in circular genomes this term can be ambiguous, so we use it here to refer specifically to the gene order as presented in Table 2) fragment was 2806 bp-long, and the downstream was 2767 bp; aligned, they were 2811 bp-long. The two fragments are highly conserved, with only 0.04 base substitutions per site between the two sequences. The difference in length was caused by the loss of 39 bases in the second fragment between positions 432 and 470 in the alignment. In the upstream fragment, the 39 bases are found in the intergenic region (73 bp) between pseudo-*nad5* and tRNA-Ala, adjacent to the latter. The high similarity between the two segments indicates that they most probably originate from rearrangement events relatively recent in evolutionary terms.

### Characteristics of duplicated genes

In the process of genomic rearrangements and/or subsequent sequence evolution several genes have lost fragments of their sequences, which is highly likely to have rendered them non-functional. We added a prefix ‘pseudo-‘to the names of those genes and did not indicate start/stop codons for them (Table 2). Among the six duplicated genes: *nad1*, *nad4*, *nad5*, *nad2*, *cytb* and *cox3*, only the latter three appear to possess two (mostly) complete copies. Both copies of *cox3* use identical codons (ATT and TAA), are of identical length (762 bp), and have almost identical sequences, apart from five SNPs at positions 13, 29 and 109, 349, 398. Intriguingly, those translate into the equal number of mutations in the amino acid sequence, which is likely to be a sign of a relaxed purifying selection conferred by the functional redundancy. Pairwise comparison with related homologs showed that the *cox3* copy (which we presume to be the original gene, Additional file 3A) appears to be the faster-evolving one. Regardless, both copies might still be functional.

Between the two *cytb* copies, the original one (*cytb*) lacks 15 bp at the 5′ end (1099 vs 1084 bp). This is an annotation artefact caused by a different start codon choice: as opposed to the 3 bp overlap between *cytb-2* and the upstream flanking tRNA-Ile-2, there is an 11 bp IGR between the *cytb* and tRNA-Ile (Table 2). Although the GTT start codon is conserved at the 3′ end of tRNA-Ile, a deletion mutation in what is now the IGR between the two genes has caused a frameshift in the ORF of *cytb*. If this is not merely a sequencing artefact, the mutation may have rendered this (original) copy non-functional, or it simply uses the next GTT triplet as the start codon (as proposed in our annotation). Comparison with homologs indicates that several of them are merely one ATT triplet longer (the preceding triplet in *C. cotti cytb* is AAT), whereas *Gnathostoma* species even lack additional six amino acids at their 5′ end. Otherwise, both sequences are relatively similar, with merely 0.04 base substitutions per site, but these translate to nine polymorphic sites between the two amino acid sequences.

Our analyses suggest that *nad2* gene is likely to be a part of a relatively large tRNA-Asp + *nad1* + tRNA-Tyr + *nad2* duplicated fragment (Additional file 3). With 0.09 base substitutions per site, the two fragments appear to be relatively highly conserved, but translated protein sequences exhibit 0.15 amino acid substitutions per site, suggesting unusually high number of non-synonymous mutations. Translated sequences revealed that the *nad2* copy, hypothesised by us to be the original gene (Additional file 3), possesses internal stop codons and thus is most probably non-functional. To reflect this, although the sequence is complete, we have added the prefix ‘pseudo-‘to its name (Table 2). At the end of the same fragment, we found a highly conserved 410 bases-long fragment of the 5′ end of *nad1* gene, including a conserved TTG start codon. The rest of the gene is highly degraded or missing. Pseudo-*nad4* is a relatively well-conserved 243 bp 5′ fragment of the gene, whereas pseudo-*nad5* is a highly degraded 3′ fragment of the gene. The pattern of conserved/deleted fragments for all three genes is in agreement with the hypothetical evolutionary history of genomic rearrangements, with deletions at the edges of the copied fragments (Additional file 3). Therefore, we hypothesise that the losses of the ends of these genes may have occurred during the rearrangement events.

Sequence duplications are non-adaptive events [47] likely to reduce the evolutionary fitness of the organism, and thus should be under an evolutionary pressure directed towards the loss of the superfluous duplicated genes and genomic regions [48], although experimental evidence indicates that this process is not always very efficient [49]. Different levels of conservation between these six duplicated genes indicate either that the speed of this process of removal of superfluous genomic regions varies between different parts, or that rearrangement events occurred over a relatively long time-period.

### Transfer RNAs

Non-standard secondary structures of tRNAs, usually lacking either a TΨC or a DHU arm, are common in nematodes [9, 13, 50, 51]. Duplicated tRNAs are also not particularly rare [10], but usually it is merely one tRNA that is duplicated, e.g. [27], whereas *C. cotti* mitogenome possesses seven pairs of duplicated tRNAs: Trp, Tyr, Ala, Asp, Gln, Ile, and Thr. On the other hand, nematodes usually possess all 22 standard tRNAs [9, 10], whereas we could identify only 19, with tRNA-Leu1^(CUN)^, Ser2^(AGN)^ and tRNA-Phe missing. Generally, tRNAs are the gene category with the highest ‘dispensability’ in the mitochondrial genome, so mitogenomes of some animals don’t possess the full set of tRNAs [10, 52], which is believed to be compensated by the import of tRNAs from the cytoplasm [53]. We can only speculate that this may be the mechanism through which *C. cotti* compensates for the absence of these three tRNAs as well.

**Fig. 2:**
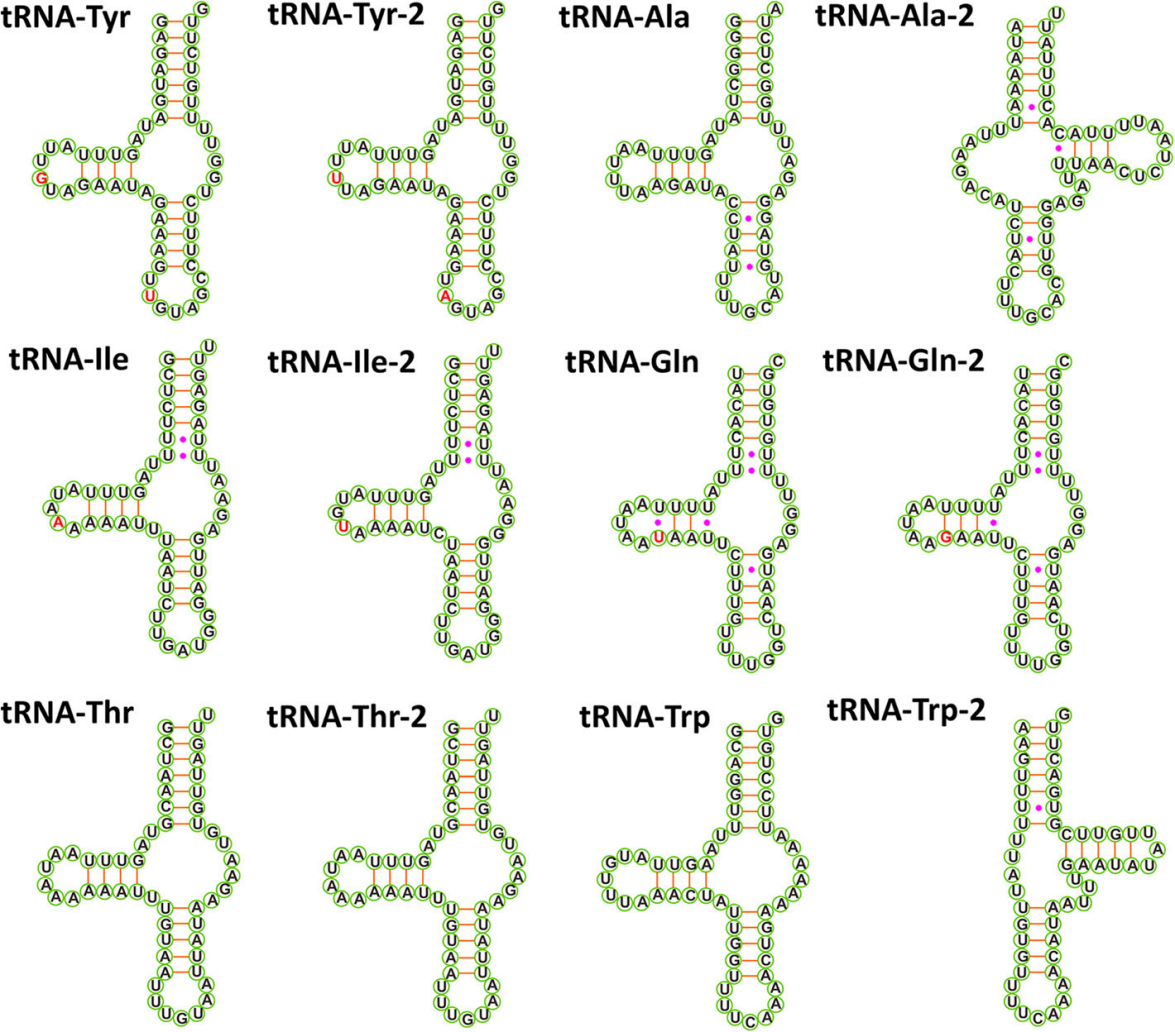
Comparison of duplicated tRNAs in the mitochondrial genome of *C. cotti*. Orange line indicates normal base pairing, lilac dot indicates non-standard base pairs, and red bases highlight where two tRNAs differ only in one base

We have used the inferred hypothetical evolutionary history of genomic architecture rearrangements (Additional file 3) to attempt to search for traces of these missing tRNAs. Based on the gene orders in related species (Fig. 1), we expected tRNA-Leu1^(CUN)^ to be found in the tRNA-Gln to *nad4* duplicated fragment, between *cytb* and *cox3* (Additional file 3). Indeed, a 44 bp-long IGR exists in both fragment copies in *C. cotti* (Table 2). Although the two IGRs are almost identical, with only G replaced with T at position 21 in the duplicated fragment, the alignment with tRNA-Leu1^(CUN)^homologs indicates low similarity, the absence of a conserved anticodon, and a large deletion at the 5′ end. The most probable position of tRNA-Phe would be downstream from tRNA-Arg, which is where we found a 47 bp IGR in *C. cotti*. The non-coding sequence did indeed exhibit similarity to related homologs, but a 6-bp deletion where the anticodon should be indicates that it has also lost its functionality. We presumed that tRNA-Ser2 should be in the place where tRNA-Trp-2 is found in the mitogenome (Additional file 3). Sequence comparison has indeed confirmed that the two tRNA-Trp genes don’t share evolutionary ancestry (Fig. 2), and that tRNA-Trp-2 is much more similar to tRNA-Ser2 homologs than to tRNA-Trp homologs. However, its anticodon base triplet has undergone a mutation from TGA to TCA. As a result, the mitogenome now possesses what appear to be two functional tRNA-Trp genes. Remolding of tRNAs is a well-documented process that involves a duplication of a tRNA gene, a mutation that changes the anticodon and the loss of the ancestral tRNA gene [54], but in this case there is no indication that tRNA-Ser2 was duplicated in the first place. Suspecting a sequencing artefact, we have checked the electropherogram, but there was no indication of poor sequencing quality either. This phenomenon should be further confirmed using a *C. cotti* specimen belonging to a different lineage.

Another indication of remolding was observed in tRNA-Val, as its sequence was very divergent from all remaining homologs, but the anticodon defined it as Valine. Comparison with other species indicates that its position is rather instable (Fig. 1). In the closely related *D. medinensis*, tRNA-Cys can be found in that position, but the sequence isn’t similar to tRNA-Cys homologs either. BLASTing of the sequence did not return any significant hits either. We can only speculate that this phenomenon might be a reflection of another tRNA remolding in the evolutionary history of this species, where a mutation in the anticodon has led to subsequent rapid evolution of the entire DNA sequence.

The status of the tRNA-Ala-2, presumed to be a part of the duplicated fragment with pseudo-*nad5* gene, is also ambiguous: its anticodon and central part of the sequence are conserved among the related homologs, but its 5′ end exhibits a 6-bp deletion. It can still be folded into a tRNA-like structure, but a very non-standard one, and very different from the tRNA-Ala copy (Fig. 2). Thus we remain doubtful regarding its functionality.

The annotation of tRNA-Lys was also ambiguous: depending on the algorithm employed, it was predicted as both Asn and Lys (ARWEN indicated it could be Lys or Asn, and MITOs that it is Asn). Comparison with homologs was also ambiguous, as the two most similar sequences appeared to be *D. medinensis* tRNA-Lys and *P. sanguineus* tRNA-Asn. As the anticodon was UUU, the tRNA was identified as Lys. Alignment with related homologs indicated that these two tRNAs (Lys and Asn) in Camallanoidea and Dracunculoidea families are generally very different from other related homologs, which suggests a possibility of yet another remolding event(s) in the common ancestor of these two families.

Although the annotation of tRNA-Pro was not ambiguous, it was the only homolog that exhibited GGA anticodon, whereas the remaining spirurids all use GGG. tRNA-Thr, tRNA-Gln and tRNA-Ile were duplicated as a part of the large tRNA-Gln - *nad4* fragment (Additional file 3). The two tRNA-Thr sequences were identical, whereas the latter two pairs of sequences both exhibited only one SNP (Fig. 2). tRNA-Tyr and tRNA-Asp were probably duplicated in a fragment also containing *nad1* and *nad2* (Additional file 3). However, possibly because tRNA-Tyr is located between the two PCGs, both copies are highly conserved, exhibiting only two SNPs and identical structure (Fig. 2). In contrast to this, one of the two tRNA-Asp copies has completely lost the 5′ part of its sequence (pseudo-tRNA-Asp in Table 2). The loss is likely to have occurred in the rearrangement process, as it is located on the edge of the copied fragment (Additional file 3). The rearrangement event is also likely to have been relatively recent in evolutionary terms, as the 33 bps adjacent to *nad2* (3′ end) still exhibit a highly conserved sequence with only two polymorphic nucleotides in comparison to the corresponding fragment of the other tRNA-Asp copy.

### Base composition and skewness

Mitogenomes of nematodes usually exhibit an A + T bias, often higher than 70% [35], and sometimes even higher than 80% [13]. Thus, despite its apparently high A + T bias of 70.8%, *C. cotti* actually exhibits the lowest bias among the available Spirurida mitogenomes, with the exception of *Gnathostoma doloresi* at 70.5% [38]. This might be a reflection of lower mutational constraints on the non-functional duplicated parts of the mitogenome. Intriguingly, in Spirurida and Tylenchida orders, this bias mostly appears to be driven by the extremely high T-content, with T base often representing more than 50% of the nucleotides [22]. Apart from the three *Gnathostoma* species and *P. sanguineus*, all available Spirurida mitogenomes exhibit T-bias values over 50%: from *C. cotti* at 51% to 56.9% in *Dirofilaria repens* (Additional file 2).

The studied mitogenome exhibits a negative AT-skew (−0.441) and a positive GC-skew (0.463). In this aspect it is also not an outlier among the available spirurid mitogenomes, which exhibit AT-skews between −0.4 and −0.5, and GC-skews between 0.35 and 0.52 (Additional file 2). In agreement with the T-base abundance findings, the three *Gnathostoma* species (−0.28, −0.29 and −0.33) and *P. sanguineus* (−0.21) exhibited lower AT-skew values. Surprisingly though, *H. longissimum*, which was not an outlier in terms of T-base abundance (52.9%), was an outlier in terms of AT-skew (−0.34). Additional intriguing observation is that the two mitogenomes belonging to the outgroup species, *L. polyphemus* and *T. spiralis*, exhibited an opposite skew trend, with positive AT-skew and negative GC-skew values.

### Overlaps between genes

The thirteeen observed overlaps ranged from 1 to 11 bp (Table 2), but we consider only five of them relatively large (≥ 5 bp): pseudo-tRNA-Ala/tRNA-Gln and *16S*/*nad3* (both 11 bp), *nad6*/*nad4l* (6 bp), and both *cox3*/tRNA-Thr segments (5 bp). The first overlap is likely to be an annotation artefact caused by the previously discussed 6-bp deletion at the 5′ end of pseudo-tRNA-Ala. Although it may appear that the *16S*/*nad3* overlap is also likely to be an annotation artefact, as the exact boundaries of rRNAs are difficult to predict and thus usually presumed to extend to adjacent genes [55], alignment of *16S* with other available spirurid homologs revealed that they are highly conserved, and that removing the overlap from the annotation would require truncating its relatively conserved 3′ end. As the *C. cotti* 16S rRNA, at 938 bp, is already the smallest reported so far, with comparable sizes observed only in Gnathostomatidae (Additional file 2), we decided to keep the overlap. Furthermore, an 8 bp-long overlap with *nad3* was also proposed in two other spirurids: *S. lupi* and *Thelazia callipaeda* [35]. The *cox3*/tRNA-Thr overlap, also perfectly preserved in the duplicated fragment, can be reduced down to only 3 bp if *cox3* uses the truncated T-- stop codon, presumed to be completed (UAA) by the addition of 3′ A residues to the mRNA [56–58]. Apart from *nad6*/*nad4l*, all of the overlaps in the studied mitogenome involve tRNA genes. This is common, and believed to be a consequence of lesser evolutionary constraints on tRNA sequences [59]. However, *nad4l* is a very small gene (234 bp in *C. cotti*), which also appears to be under lesser evolutionary constraints as mitogenomes of some groups of animals often exhibit overlaps involving this gene [60, 61]. Thus we don’t deem this overlap particularly suspicious. A large number of gene overlaps in mitochondrial genomes of metazoans are believed to be a reflection of strong selection for small size [19]. Although it may appear incongruous to observe as many as 13 gene overlaps in a mitogenome carrying such a large number of (presumably) unnecessary duplicated genes, they are most probably merely a remnant of a more stable (and compact) phase in the evolutionary history of this mitogenome.

### Protein-coding genes: Length and codons

Gene lengths among the available Spirurida mitogenomes were not very conserved, with four genes exhibiting a difference of more than 50 bp between the largest and the smallest compared gene: *nad3* was the most conserved with only 16 bp and *nad2* the least with 228 bp (Additional file 2). None of the *C. cotti* PCGs were major outliers regarding the size. Most of the genes used the two start (8 TTG + 3 ATT) and three stop (4 TAG, 3 TAA, 7 T--) codons common for nematodes [9, 13, 20, 27]. The third observed start codon, GTT for *cytb* (and *cytb-2*) and *nad4*, was also observed in a number of closely related nematodes [38, 62, 63]. The length of *nad4* remains ambiguous: we have selected the length of 1233 bp, identical to some *Onchocerca* species (Additional file 2), in which case there are no intergenic nucleotides between tRNA-Thr and *nad4*. On the other hand, as the 5′ end of the *nad4* gene is GTTTTTTATGTTCTGTTT, the second GTT may also be used as the start codon, in which case there would be nine intergenic nucleotides and *nad4* gene would be 1224 bp long, which is identical to *G. doloresi*.

### Non-coding regions

The AT-rich region, believed to function as the control region containing the replication origin [64], is usually located between *nad4* and tRNA-Met in mitogenomes of other nematodes [7]. In *C. cotti* these two genes are not adjacent and the AT-rich (237 bp) is located between tRNA-His and tRNA-Arg (Table 2). Notwithstanding the AT-rich, IGRs found in the mitogenome ranged from 1 to 279 bp in length (21 in total). These numbers are larger (both the total number and maximum size) than reported in several closely related Spirurida mitogenomes: 14–16 IGRs, 1 to 62 bp [32, 35], which is not unexpected for a mitogenome exhibiting such a large number of rearrangements.

### Evolution of mitogenomic arschitecture

Mitogenomic gene rearrangements should be strongly selected against, as they can affect the replication and transcription mechanisms, and produce disruptions in the gene expression co-regulation [65]. In agreement with this, although mitogenomes of nematodes exhibit exceptional variability in the mitogenomic architecture, most of it is found in several families within the class Enoplea, whereas Chromadorea (containing the majority of Nematoda species) exhibit very few gene rearrangements [2, 7, 9, 10, 27, 43, 46, 66]. Frequent sequence rearrangements and long sequence duplications comparable to the ones found in the *C. cotti* mitogenome have been observed only in nematodes belonging to the Mermithidae family (Enoplea). Several species within this family exhibit hypervariability in conspecific size variation in mitogenome size (19–34 Kb), owing to long duplicated fragments of several Kb, containing both PCGs and tRNAs, present in different copy numbers within individual mitochondrial genomes [45, 46, 67]. The proposed mechanism explaining this hypervariability is mitogenomic recombination [68, 69]. Interspecific mitochondrial recombination has been observed in several animal lineages aside nematodes, including fishes [70] and birds [42], but it is believed to be accompanied by mitochondrial gene inversions (frequent exchange of the coding strand) [10]. Characteristics of the *C. cotti* mitogenome, especially the encoding of all genes on a single strand and almost identical sequences between some of the duplicated genes, suggest that interspecific recombination is an unlikely scenario. The most common mechanism of mitogenomic architecture rearrangements is believed to be slipped-strand mispairing mechanism leading to a tandem duplication and subsequent evolutionary loss of duplicated genes (TDRL) [10, 48, 49, 65, 71, 72]. However, the observed architecture and the conducted CREX analysis imply that the mechanism would require several duplications of the entire genome followed by extensive losses (Supplementary file 3C). As we did not observe traces of the latter process in the mitogenome (we would expect it to produce a much larger number of NCRs), we believe that transposals and duplications followed by transposals of duplicated fragments are a more parsimonious explanation for the observed mitogenomic architecture rearrangements (Additional file 3, A and B). The evolution of gene orders mostly driven by transpositions has been proposed in ascomycetes as well [73]. A fourth hypothesis would be fragmentation of the “standard” single-circle mitogenome into multipartite genomes, followed by subsequent re-organisation into a single circular molecule (we did not find any indications that the mitogenome is fragmented in *C. cotti*). Mitogenome fragmentation is common in higher plants [74, 75], and has occurred independently in some metazoans, including mesozoans [76], cnidaria [77], insects [78], rotifers [79, 80], as well as nematodes [21, 81]. A very similar scenario, preceded by an ancestral mitogenome duplication, has been proposed before for another nematode, *Globodera ellingtonae* [81]. Although the relative abundance of pseudogenes is in agreement with this course of events [81, 82] and a single genome duplication is more parsimonious than a series of duplications required for the TDRL process, it remains hypothetical.

Regardless of the exact rearrangement mechanism, mitogenomic architecture of *C. cotti* is unique not only among Chromadorea, but also among almost all nematodes. Although it is believed that lengthy sequence duplications are absent from mitogenomes of chromadorean nematodes [46] and that only rearrangements in tRNA order are common [27], our analysis shows that neither of the propositions is true for the families clustering in the Camallanina clade (as defined in this study). As gene order instability has been observed in another chromadorean order, Oxyurida [66], this hints towards a possibility that some chromadorean orders (or more precisely specific groups of families within them) might be particularly prone to mitogenomic rearrangements.

Based on the observation of high variability in mitogenomic architecture in several phylogenetically distant groups, it has been proposed that the acceleration of the rate of genomic rearrangements in the evolutionary history of metazoans has occurred independently on several occasions [10]. It is known that mitochondrial genomes of some lineages of vertebrates are evolutionary dynamic even at short timescales [83, 84]. For example, different duplication mechanisms were observed in highly dynamic mitogenomes of some salamanders [84]. As genomic rearrangements are random evolutionary events [72], not driven by positive selection [47], and as there is evidence for positive correlation between compactness of genomes and structural stability [85], there is mounting evidence that the evolution of mitogenomic architecture is highly discontinuous. Therefore we hypothesise that, although mitogenomic rearrangements are generally strongly selected against, which results in long evolutionary periods of stasis in gene content and arrangement in most metazoan lineages, once a rearrangement event has destabilized the genomic architecture, this is likely to be followed by an exponentially accelerated rate of mitogenomic rearrangements.

## Conclusions

We have sequenced and characterised the complete mitochondrial genome of the fish-parasitising chromadorean nematode *Camallanus cotti*. It is exceptionally large among chromadoreans and exhibits a unique architecture, with a large number of duplicated genes (46 genes and pseudogenes were identified in the mitogenome). Among the six duplicated PCGs, three were incomplete, and one contained stop codons in its sequence; thus only two pairs are likely to be functional. Among the six duplicated tRNAs, five still appear to possess two functional copies. Regardless of the unusually large number of tRNAs found, only 19 unique ones were found in the mitogenome. A remolding event explains one missing tRNA, whereas intergenic regions provide clues for the other two tRNAs missing from the mitogenome. Although TDRL is considered to be the most common mechanism for gene order rearrangements, the most parsimonious explanation for the architecture observed would be either TDRL accompanied by transposals, or an even more unorthodox duplication-fragmentation-reassembly scenario.

Based on the observed rate of mitogenomic architecture rearrangements in Spirurida, as well as the evidence from other metazoans, we put forward a hypothesis that the evolution of mitogenomic architecture is highly discontinuous: once a long period of stasis in gene order and content has been punctuated by a rearrangement event, such a destabilised mitogenome is much more likely to undergo subsequent rearrangement events, resulting in an exponentially accelerated evolutionary rate of mitogenomic rearrangements. This hypothesis still needs to be tested using a large number of mitogenomes and appropriate statistical methods, but the implications of this model are particularly important for the gene order similarity analyses, which have often been used as an additive source of phylogenetic information for Chromadorea class [2, 10, 27, 43, 46, 66]. The mounting evidence for discontinuous evolution of mitogenomic order in this class indicates that the aforementioned approach is too prone to overinflated estimates of evolutionary distance to have practical usability.

Chromadorean nematodes might be a good model to further study this discontinuity in the dynamics of mitogenomic evolution, as the class is comprised mostly of orders exhibiting extreme (for nematodes) stability of mitogenomic architecture, interrupted by clades of taxa that exhibit varying degrees of accelerated mitogenomic architecture evolution. As *C. cotti* possesses a mitogenome exhibiting an extremely accelerated rate of rearrangements, future studies should aim to sequence more mitogenomes belonging to families comprising the Camallanina clade, with particular focus on the Camallanidae family.

## Methods

### Sampling

It is known from a previous study that Chinese hooksnout carp (*Opsariichthys bidens* Günther, 1873) is particularly susceptible to *C. cotti* infections, with mean prevalence of almost 50% in wild populations from a large reservoir in central China, Danjiangkou (32°82′50″ – 33°81′50″ N; 11°08′70″ – 11°18′60″ E) [86]. This is most probably in the native range of *C. cotti* [24]. Parasitic nematodes were obtained post mortem from hooksnout carp specimens caught by fishermen in the Danjiangkou and bought from the local market on 23/Apr/2016. Live nematodes were removed from the fish intestines, and then taxonomically identified by their morphological characteristics via dissecting microscopy, using several sources as guidance [25, 87, 88]. All nematodes were washed in 0.6% saline before being stored in absolute ethanol in the Museum of Aquatic Organisms, Institute of Hydrobiology, Chinese Academy of Sciences, Wuhan, China (Accession No. IHB20160324005).

### Genome sequencing and assembly

Genome was sequenced broadly following the procedure described before [60, 80]. After soaking a single adult nematode (≈3.5 cm) in TE buffer (pH 8.0) overnight to remove the ethanol, total genomic DNA was extracted using the Aidlab DNA extraction kit (Aidlab Biotechnologies, Beijing). Eight degenerate primer pairs were designed (Table 3) to match the generally conserved regions of mtDNA genes and used to amplify and sequence short fragments of 12S, *cytb, nad4, nad1, cox1*, *cox2*, 16S and *nad5* genes. These sequences were then used to design 12 specific primers for the amplification and sequencing of the remaining mitogenomic sequences in several PCR steps. Primers were designed to produce amplicons with overlaps of about 100 bp. Reaction volume of 50 μL contained 5 U/μL of TaKaRa LA Taq polymerase (TaKaRa, Japan), 10 × LATaq Buffer II, 2.5 μM of dNTP mixture, 0.2–1.0 μM of each primer, 60 ng of DNA template, and PCR-grade H_2_O. PCR conditions were optimized for each reaction, with the annealing temperature adjusted to suit the specific primer pair, extension time set to 1 min per Kb of the expected product size, and cycles (35 on average) adjusted depending on the amplification efficiency of primers. PCR products were sequenced on an ABI 3730 automatic sequencer using Sanger method [89]. When problems in sequencing were encountered, products were cloned into a pMD18-T vector (TaKaRa, Japan) and then sequenced. All obtained fragments were quality-proofed (electropherogram) and BLASTed [90] to confirm that the amplicon is the actual target sequence. Whenever the quality was sub-optimal, sequencing was repeated. Mitogenome was assembled stepwise with the help of DNAstar v7.1 [91] program, making sure that the overlaps were identical, and that no *numt*s [28] were incorporated into the sequence. After it became obvious that the architecture of this mitogenome is very unusual, to further confirm that this is not a consequence of PCR artefacts, DNA pollution, *numt*s, or a feature unique to the specimen from which the DNA was extracted, we have extracted DNA from another specimen (sampled as described above from the same fish specimen), and repeated the procedure using long-range PCR with the specific primers (Table 3).

**Table 3:** Primers used for amplification and sequencing of the mitochondrial genome of *C. cotti*

Gene span	Primer name	Sequence (5′-3′)
nad6-12S	DYF1	TTGCGGTGCTTTGCGTTCTG
	DYR1	CTCTCGTTTAACAGTCAACC
12S	XW12SF	GTTCCAGATTAATCGGCTA
	XW12SR	CAATTGATGGATGATTTGTACC
12S–cytb	DYF2	CACGAGTTTAGGTTGAGCCAC
	DYR2	CAACGTAGAGCAAGAAACC
cytb	XWCYTBF	GRGCTCARATGAADTATTGAGC
	XWCYTBR	TATCACTCTGGCACHAYATG
cytb-nad4	DYF3	CCTTTTCTGATGGGGGATCC
	DYR3	GAACACAACACAGCACCCAA
nad4	XWND4F	GCYCATGTTGARGCACCTAC
	XWND4R	GAAGAATARGCAGCTAAWG
nad4-nad1	DYF4	GTTATTGTCGTTTTTGGGTGC
	DYR4	GGGTAATACAAATCACAACATCAG
nad1	XWND1F	GCGTRTTGGTCCTAAYAAGG
	XWND1R	TATCAWAACGGWAACGTGGG
nad1-cox1	DYF5	GTGTTCCTTTTGTTCTACTC
	DYR5	GAACTTCCTGACAAACAACTAC
cox1	XWCOX1F	ATYGGTGGATTYGGWAATTG
	XWCOX1R	TAAACCTCNGGGTGHCCAA
cox1-cox2	DYF6	ACTATGTTGTTACTAGATCG
	DYR6	CCACAAGGCACCACACAACG
cox2	XWCOX2F	CTGAACTTATGATTACAGA
	XWCOX2R	CCACAAATCTCWGAACAYTGACC
cox2-16S	DYF7	GGCAGATGCTTTGTCTGGGG
	DYR7	TTCCGAAGACTTATCTTTG
16S	XW16SF	ATGGCTGYTWTAGCGTGAGG
	XW16SR	TCTATCTCACGAYGAAYTAAAC
16S–nad5	DYF8	GTAGTTCTTATACATTTTCAGG
	DYR8	ATGGTCAACAGACCAACA
nad5	XWND5F	TAGCYTTAGGYAATCACCTAC
	XWND5R	GAGACAWGGTNCTCAAWGCCAC
nad5	DYF9	GGCTTTTTGTTTTTCTGATG
	DYR9	CTACATGCAAAATAGGTATC
nad5-cytb-2	DYF10	GGTGCTGGATAGTTATTCTGG
	DYR10	CAATCAACTCAAAACAACAG
cytb-2-nad6	DYF11	GTTCTGTTCCTAGGAAGATC
	DYR11	CCACAAAAACTCAACAAAGGAC
nad6	DYF12	CTATGTTTCGCTTACAACG
	DYF13	CTACTCATAACACAAAAAC

Primer names beginning with XW indicate a generic primer, whereas DY indicates a specific primer pair

For reasons we only partially understand, amplification and sequencing of this mitogenome was a comparatively difficult and laborious process, wherein obtaining both good quality PCR and sequencing results was not a straightforward process, especially for the duplicated segments. This was observed before for helminths, and suspected to be a result of high A + T content [92]. Further elements contributing to this may include the low amount of DNA that can be obtained from a single nematode, or even complex quaternary structure of the sequences.

### Annotation

The mitogenome was annotated broadly following the procedure described before [61, 93]. Protein-coding genes were found by searching for ORFs (employing genetic code 5, invertebrate mitochondrial) and alignment against the adopted reference genome (*D. medinensis*) in Geneious [94]. Each gene was then aligned with homologous sequences of closely related species (Table 1) and annotation fine-tuned manually. The two ribosomal RNAs were annotated in a similar way, via comparison with homologs. MITOS [95] and ARWEN [96] tools were used to detect and fold the tRNAs in the genome. Folded tRNAs were further prepared for publication using Coreldraw (Corel Corp.). Because tRNAs of nematodes often exhibit non-standard secondary structures [50], after the automatic annotation by these algorithms, all non-coding regions larger than 30 bp were carefully examined for the existence of tRNA-like sequences. Ambiguous tRNAs were additionally visually aligned with homologs extracted from all analysed mitogenomes using a custom-made GUI-based program MitoTool [97]. Annotation of tRNAs proved to very ambiguous, mostly as a result of many tRNA genes with non-standard sequences and/or secondary structures. We have tweaked the annotation several times, but we remain non-confident that it is fully accurate. MitoTool was also used to extract the annotation manually recorded in a Word document and to generate tables with statistics for all analysed mitogenomes. Pairwise distances were computed using MEGA 7 [98]. NCBI’s Organelle Genome Resources were used to compare it with other published mitogenomes.

### Phylogenetic and gene order analyses

Following the proposed guidelines for new mitogenome validation [30], we have conducted a barcoding identification assessment and a mitochondrial phylogenomic analysis using almost the entire new mitogenome. For the former, all 99 Camallanidae sequences were retrieved from the BOLD database [31]. Sequences were aligned by MAFFT [99], and raxmlGUI [100, 101] used to conduct maximum-likelihood phylogenetic analysis with 1000 bootstrap replications. For the phylogenomic analyses, all 21 available (June 2017) unique Spirurida mitogenomes were retrieved from the GenBank. It should be noted that sometimes Camallanida are elevated to a status of order [102], but the GenBank classification system includes them in Spirurida. Preliminary comparisons of mitogenomes retrieved from GenBank have indicated that *Gongylonema pulchrum* (NC_026687) [103] *nad4* gene was misannotated. Hence, in order to be able to conduct phylogenetic and gene comparison analyses, we have re-annotated the gene by moving the start codon 15 bases in 5′ direction, and changing it from ATT to GTT, thus creating an overlap with the adjacent tRNA-Thr. In all analyses, two sequences were used as outgroups: a basal nematode [27, 43] *T. spiralis* (NC_002681) [50] and a basal arthropod *L. polyphemus* (NC_003057) [43, 104]. Complete mitogenomic sequences selected for the analysis were retrieved from the GenBank, genes extracted using MitoTool, and further handled with another custom-made GUI-based program, BioSuite [105]. Phylogenetic analyses were performed using concatenated 33 genes: 12 PCGs (*atp8* missing), 2 rRNAs and 19 tRNAs (three missing from *C. cotti*). For duplicated genes, the one presumed to be functional, or original where both seemed to be functional (see Table 2 and discussion for details), were used. Each sequence was aligned separately (in batches) by MAFFT integrated into BioSuite: rRNAs and tRNAs were aligned using normal alignment mode, whereas codon-alignment mode was used for PCGs. Finally, BioSuite was used to concatenate the alignments and produce input files for the two programs used to conduct the phylogenetic analysis. Maximum-likelihood analysis (with 1000 bootstrap replications) was conducted using raxmlGUI, and Bayesian inference using MrBayes 3.2.6 [106] with default settings and 5 × 10^6^ generations (average SD of split frequencies = 0.000054). GTR + G + I evolution model, selected using ModelGenerator [107], was used in both analyses. Saturation analysis results, conducted using DAMBE program [108], indicate low saturation for all three codon positions. Finally, phylograms and gene orders were visualised and annotated by iTOL [109] with the help of several dataset files generated by MitoTool.

To make it easier for the readers to visualise the extent of rearrangements in the Camallanina clade, we present some of the hypothetical rearrangement scenarios in the Additional file 3. However, these results should be interpreted with caution. Using the obtained BI phylogram, we conducted an ancestral character gene order state reconstruction using MLGO web server [110]. None of the available tools to infer genome rearrangements (among those we could find) could handle mitogenomes with both gene deletions and duplications. Therefore, we have inferred the approximate number of gene order rearrangements needed between *Camallanus cotti* and ancestral node A1, and shown them in the Additional file 3A. As the putative gene orders of the ancestral nodes A2 and A1 (as defined in Fig. 1) inferred by MLGO did not contain duplicated genes, CREX algorithm [111] was used to infer the hypothetical evolutionary history of gene order rearrangements (Additional file 3C). However, the results suggest three duplications of the entire mitogenome followed by a random loss of genes. As we could not find evidences supporting this scenario, i.e. numerous NCRs, we are skeptical about this result. Therefore, we have also visualised (Additional file 3B) the extent of gene order rearrangements between the two nodes following the same logic as in the Additional file 3A.

## Additional files

Additional file 1: Taxonomic identification of the studied *C. cotti* nematode using *cox1* barcoding. Maximum likelihood analysis was conducted on 100 Camallanidae *cox1* sequences, with GenBank accession numbers shown in the figure. The clade containing the queried sequence (‘Camallanus cotti this study’) is shaded purple. (PDF 40 kb)

Additional file 2: Statistics and gene features of Spirurida mitogenomes. Worksheet A lists all the species, GenBank accession numbers, genome sizes in bp, base composition and skew. Worksheet B lists gene sizes for all species, and their corresponding (putative) start and terminal codons. Species are represented by acronyms of their binomial scientific names. (XLSX 18 kb)

Additional file 3: Mitogenomic gene order rearrangements in the Camallanina suborder. (A). A hypothetical outline of gene order rearrangements between *Camallanus cotti* and ancestral node A1. Ancestral node A1 is shown in Fig. 1. Genes and mitogenome fragments presumed to have undergone rearrangements are highlighted by different colours. Hypothetical rearrangement mechanism is indicated on the left, and arrows are used to indicate the putative translocation pathways. Where the presumed mechanism is duplication + transposal, arrows indicate the positions of both fragments in the downstream genome, where the fragment presumed to be translocated is labelled with the letter C. Where colouring is insufficiently unambiguous, fragments undergoing a rearrangement event are additionally underlined. The order in which the events are shown is (mostly) random. A star is used to indicate a deletion and a thick blue arrow to indicate a tRNA remolding event. (B) A hypothetical outline of gene order rearrangements between ancestral nodes A2 and A1. Ancestral nodes are shown in Fig. 1. Genes and mitogenome fragments presumed to have undergone rearrangements are highlighted by different colours. Hypothetical rearrangement mechanism is indicated on the left, and arrows are used to indicate the putative translocation pathways. Where colouring is insufficiently unambiguous, fragments undergoing a rearrangement event are additionally underlined. The order in which the events are shown is (mostly) random. MLGO was used to infer the putative ancestral gene orders. (C). Hypothetical evolutionary history of gene order rearrangements between ancestral nodes A2 and A1 inferred by CREX algorithm. Ancestral nodes are shown in Fig. 1. Tdrl indicates a tandem duplication and random loss event, where a duplication event (not shown) is followed by the loss of some elements (orange-shaded). This results in the remaining copies (blue-shaded) being moved to the front. (PDF 514 kb)

## Abbreviations

Bp: Base pair
IGR: Intergenic region
Kb: Kilobase
LBA: Long branch attraction
ORF: Open reading frame
PCGs: Protein-coding genes
SD: Standard deviation
SNP: Single-nucleotide polymorphism
TDRL: Tandem duplication and random loss
