# Supplementary figures and images for: The complete mitochondrial genome of parasitic nematode Camallanus cotti: extreme discontinuity in the rate of mitogenomic architecture evolution within the Chromadorea class
Source: BMC Genomics. 2017 Nov 2;18:840. doi: 10.1186/s12864-017-4237-x (PMC5669012; doi:10.1186/s12864-017-4237-x)

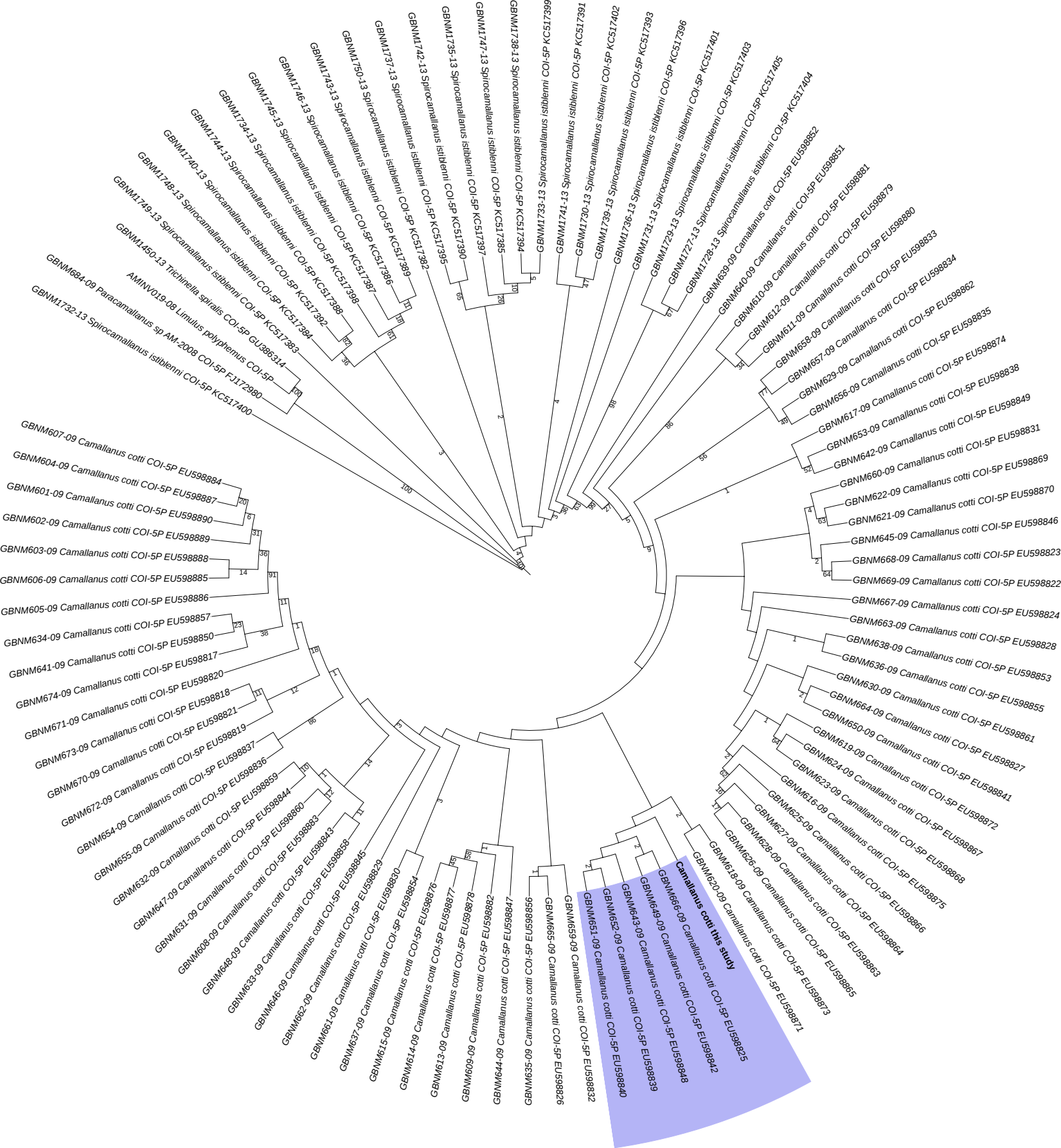

Supplement: Supplementary file 1 — Taxonomic identification of the studied C. cotti nematode using cox1 barcoding. Maximum likelihood analysis was conducted on 100 Camallanidae cox1 sequences, with GenBank accession numbers shown in the figure. The clade containing the queried sequence (‘Camallanus cotti this study’) is shaded purple. (PDF 40 kb) [file 12864_2017_4237_MOESM1_ESM.pdf]
