# Supplementary material for: The complete mitochondrial genome of parasitic nematode Camallanus cotti: extreme discontinuity in the rate of mitogenomic architecture evolution within the Chromadorea class
Source: BMC Genomics. 2017 Nov 2;18:840. doi: 10.1186/s12864-017-4237-x (PMC5669012; doi:10.1186/s12864-017-4237-x)
Supplement: Supplementary file 3 — Mitogenomic gene order rearrangements in the Camallanina suborder. (A). A hypothetical outline of gene order rearrangements between Camallanus cotti and ancestral node A1. Ancestral node A1 is shown in Fig. 1. Genes and mitogenome fragments presumed to have undergone rearrangements are highlighted by different colours. Hypothetical rearrangement mechanism is indicated on the left, and arrows are used to indicate the putative translocation pathways. Where the presumed mechanism is duplication + transposal, arrows indicate the positions of both fragments in the downstream genome, where the fragment presumed to be translocated is labelled with the letter C. Where colouring is insufficiently unambiguous, fragments undergoing a rearrangement event are additionally underlined. The order in which the events are shown is (mostly) random. A star is used to indicate a deletion and a thick blue arrow to indicate a tRNA remolding event. (B) A hypothetical outline of gene order rearrangements between ancestral nodes A2 and A1. Ancestral nodes are shown in Fig. 1. Genes and mitogenome fragments presumed to have undergone rearrangements are highlighted by different colours. Hypothetical rearrangement mechanism is indicated on the left, and arrows are used to indicate the putative translocation pathways. Where colouring is insufficiently unambiguous, fragments undergoing a rearrangement event are additionally underlined. The order in which the events are shown is (mostly) random. MLGO was used to infer the putative ancestral gene orders. (C). Hypothetical evolutionary history of gene order rearrangements between ancestral nodes A2 and A1 inferred by CREX algorithm. Ancestral nodes are shown in Fig. 1. Tdrl indicates a tandem duplication and random loss event, where a duplication event (not shown) is followed by the loss of some elements (orange-shaded). This results in the remaining copies (blue-shaded) being moved to the front. (PDF 514 kb) [file 12864_2017_4237_MOESM3_ESM.pdf]

# Additional file 3. Mitogenomic gene order rearrangements in the Camallanina suborder

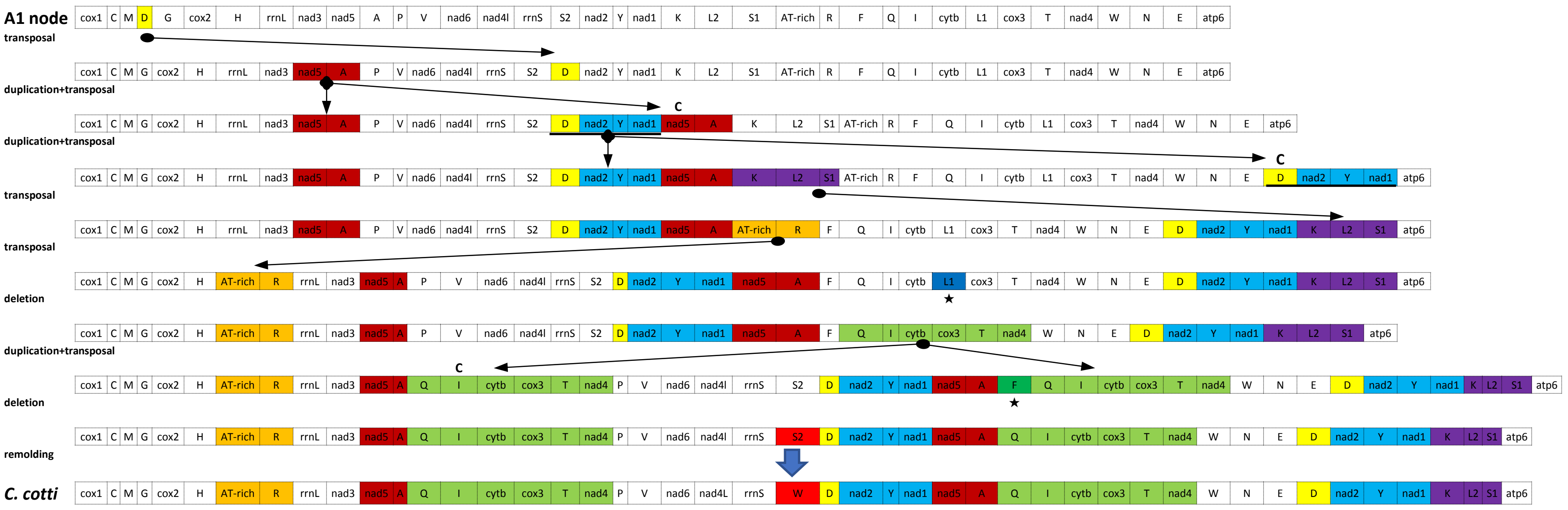

**Additional file 3A. A hypothetical outline of gene order rearrangements between *Camallanus cotti* and ancestral node A1**

Ancestral node A1 is shown in Figure 1. Genes and mitogenome fragments presumed to have undergone rearrangements are highlighted by different colours. Hypothetical rearrangement mechanism is indicated on the left, and arrows are used to indicate the putative translocation pathways. Where the presumed mechanism is duplication+transposal, arrows indicate the positions of both fragments in the downstream genome, where the putative translocated fragment is labelled with the letter C. Where colouring is insufficiently unambiguous, fragments undergoing a rearrangement event are additionally underlined. The order in which the events are shown is (mostly) random. A star is used to indicate a deletion and a thick blue arrow to indicate a tRNA remolding event.

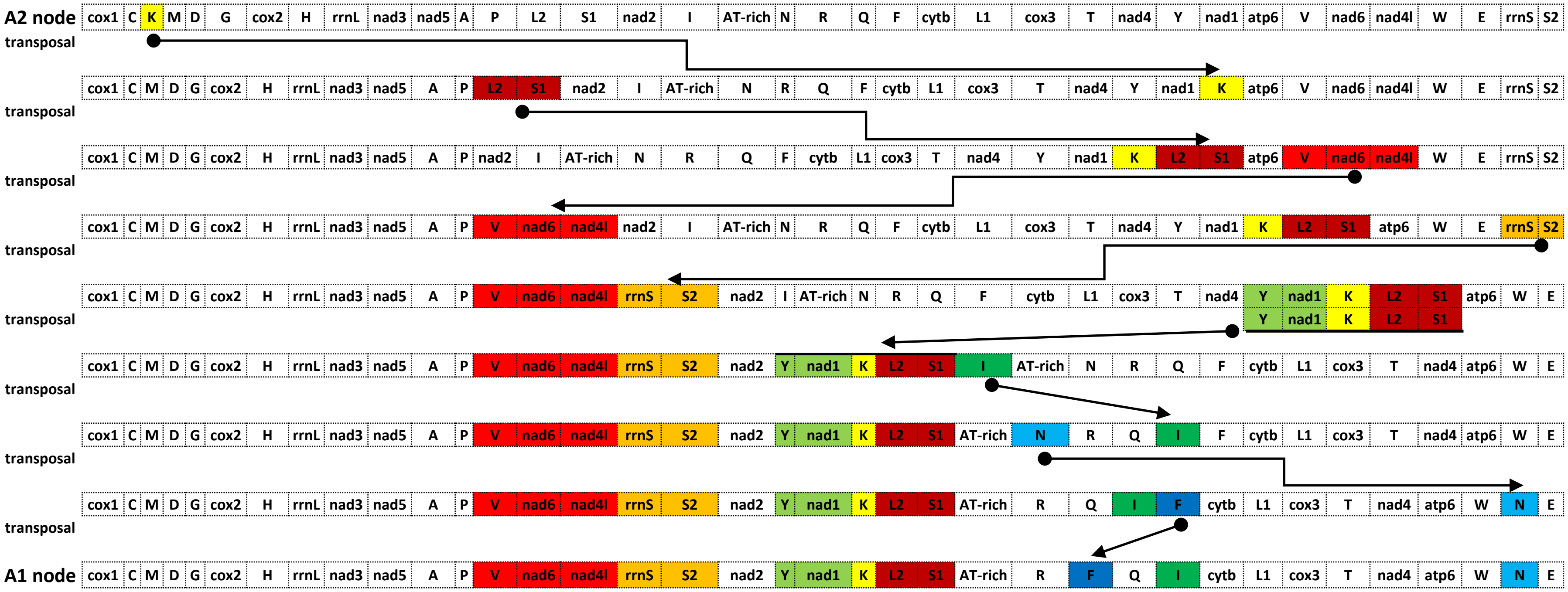


**Additional file 3B. A hypothetical outline of gene order rearrangements between ancestral nodes A2 and A1**

Ancestral nodes are shown in Figure 1. Genes and mitogenome fragments presumed to have undergone rearrangements are highlighted by different colours. Hypothetical rearrangement mechanism is indicated on the left, and arrows are used to indicate the putative translocation pathways. Where colouring is insufficiently unambiguous, fragments undergoing a rearrangement event are additionally underlined. The order in which the events are shown is (mostly) random. MLGO was used to infer the putative ancestral gene orders.

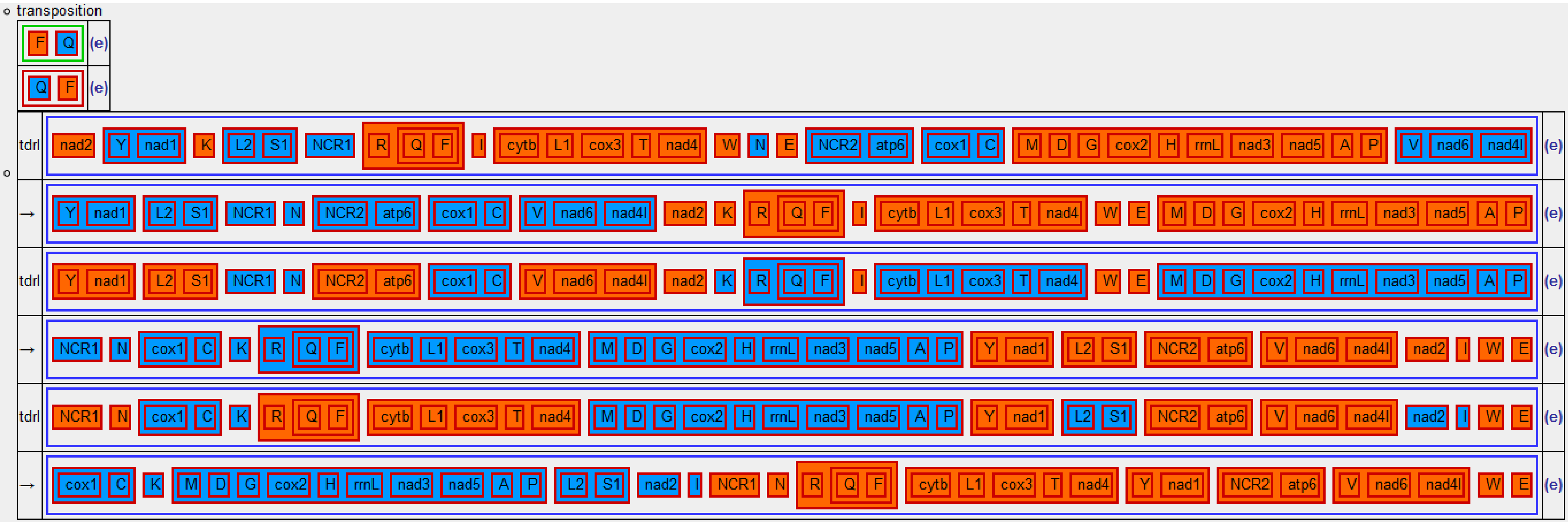


**Additional file 3C. Hypothetical evolutionary history of gene order rearrangements between ancestral nodes A2 and A1 inferred by CREX algorithm**

Ancestral nodes are shown in Figure 1. Tdrl indicates a tandem-duplication-random-loss event, where a duplication event (not shown) is followed by the loss of some elements (orange-shaded). This results in the remaining copies (blue-shaded) being moved to the front.

The complete mitochondrial genome of parasitic nematode *Camallanus cotti*: extreme discontinuity in the rate of mitogenomic architecture evolution within the Chromadorea class. Hong Zou, Ivan Jakovlić, Rong Chen, Dong Zhang, Jin Zhang, Wen-Xiang Li *, Gui-Tang Wang. BMC Genomics 2017. *Corresponding author: liwx@ihb.ac.cn (WXL)
